# CTCF and ncRNA Regulate the Three-Dimensional Structure of Antigen Receptor Loci to Facilitate V(D)J Recombination

**DOI:** 10.3389/fimmu.2014.00049

**Published:** 2014-02-11

**Authors:** Nancy M. Choi, Ann J. Feeney

**Affiliations:** ^1^Department of Immunology and Microbial Science, The Scripps Research Institute, La Jolla, CA, USA

**Keywords:** V(D)J recombination, antigen receptor, chromatin, non-coding RNA, CTCF, histone modification, chromatin loop

## Abstract

At both the immunoglobulin heavy and kappa light chain loci, there are >100 functional variable (V) genes spread over >2 Mb that must move into close proximity in 3D space to the (D)J genes to create a diverse repertoire of antibodies. Similar events take place at the T cell receptor (TCR) loci to create a wide repertoire of TCRs. In this review, we will discuss the role of CTCF in forming rosette-like structures at the antigen receptor (AgR) loci, and the varied roles it plays in alternately facilitating and repressing V(D)J rearrangements. In addition, non-coding RNAs, also known as germline transcription, can shape the 3D configuration of the *Igh* locus, and presumably that of the other AgR loci. At the *Igh* locus, this could occur by gathering the regions being transcribed in the V_H_ locus into the same transcription factory where Iμ is being transcribed. Since the Iμ promoter, Eμ, is adjacent to the DJ_H_ rearrangement to which one V gene will ultimately rearrange, the process of germline transcription itself, prominent in the distal half of the V_H_ locus, may play an important and direct role in locus compaction. Finally, we will discuss the impact of the transcriptional and epigenetic landscape of the *Igh* locus on V_H_ gene rearrangement frequencies.

## Introduction

Antigen receptor (AgR) loci are facing a uniquely difficult task to produce a great diversity of receptors in order to recognize the limitless possibility of antigens present in the environment of an organism. With the advent of next generation sequencing, we can now determine the actual diversity of AgRs by sequencing all of the rearrangements from developing B and T cells. This diversity is created through the combinatorial recombination of multiple variable (V), diversity (D), and joining (J) gene segments at AgR loci by the RAG1/2 recombinase complex, along with the extensive junctional diversity at the V–D, D–J, and V–J junctions.

**Figure 1:**
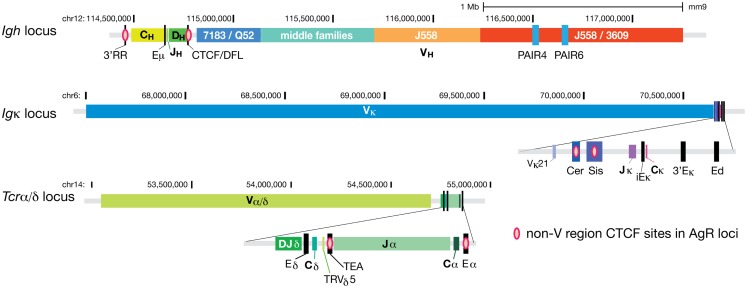
**CTCF binds at regulatory elements within AgR loci**. CTCF binding sites at all regions except for the V gene segment part of the loci for the three larger AgR loci; *Igh, Igκ*, and *Tcrα/δ*. Pink ovals represent the non-V region CTCF sites. The V gene portions of these three AgR loci have numerous CTCF sites scattered throughout the loci, hence too many to be represented. The two most prominent promoters of non-coding RNA transcribing regions of the *Igh* locus, PAIR4 and PAIR6, are also depicted as blue rectangles within the distal J558/3609 region.

One of the most extensively studied AgR loci is the mouse *Igh* locus where the V_H_, D_H_, and J_H_ gene segments span a region of ~2.8 Mb (Figure 1). The 8–13 D_H_ genes, the four J_H_ genes, and all of the constant region genes and enhancers are located within a relatively small 300 kb region. In contrast, the 195 V_H_ genes, of which ~100 were deemed to be functional, are spread out over ~2.5 Mb. To create the greatest combinatorial diversity, all V genes would have to be able to access the D_H_ and J_H_ genes relatively equally regardless of their genomic distance. The question is then, how is this equality achieved?

With growing appreciation for how three-dimensional structural changes at the locus may bring V genes into proximity of the (D)J rearrangement to which one V gene will ultimately rearrange, current studies are employing cutting edge technologies to further understand this process. Chromatin conformation capture (3C) and its more recent modifications, 4C, 5C, and Hi-C (1–3), have allowed the identification of long-range chromosomal interactions, which facilitate the rearrangement of distant V genes by making critical connections between the V genes and enhancers downstream (4). Next generation sequencing technologies coupled with chromatin immunoprecipitation (ChIP) (ChIP-seq) have allowed us to determine the binding sites of transcription factors throughout the genome as well as the genome-wide epigenetic landscape. Deep sequencing of RNA reveals the entire transcriptional profile of cells for both coding and non-coding RNA (ncRNA). Together, these techniques supply us with a bounty of information regarding the transcriptional and epigenetic profile of AgR loci at varying stages of differentiation. In this review, we will summarize and discuss how these recent studies have advanced our understanding of how long-range chromatin interactions and epigenetic changes may regulate V(D)J recombination at mouse AgR loci.

## AgR Loci Undergo Large Scale Three-Dimensional Conformational Changes during V(D)J Rearrangement

All B cell and T cell receptor (BCR, TCR) subunits are formed through the process of V(D)J recombination. The BCR consists of two immunoglobulin heavy chains (Igh) and two identical light chains encoded by either the kappa (Igκ) or lambda (Igλ) loci. The TCR alpha (Tcrα) and beta (Tcrβ), or delta (Tcrδ) and gamma (Tcrγ) chains constitute the TCR complex of the two major T cell subsets. The *Igh* and *Igκ* are of similarly large sizes of approximately 2.8 and 3.2 Mb, while the *Tcrα/δ* and *Tcrβ* loci are smaller at 1.7 and 0.66 Mb. In comparison, the *Igλ* and *Tcrγ* loci are much smaller, each only being about 200 kb. The challenge, which is particularly great for the large receptor loci, is to give all V genes a chance to undergo rearrangement in order to create a diverse repertoire. How an AgR locus brings the V genes into proximity to the (D)J genes to create this diversity is still an unanswered question.

The original observations that showed three-dimensional structural changes at the *Igh* locus, presumably facilitating the creation of a diverse AgR repertoire, came from fluorescent *in situ* hybridization (FISH) studies (5). It was found that the *Igh* and *Igκ* loci were predominantly located at the periphery of the nucleus in non-recombining cell types, but were found in more centralized locations in B cells. The nuclear periphery is generally considered a transcriptionally silent environment and is associated with repressive chromatin modifications, whereas gene dense active regions of the genome are more centrally located (6). Using two colors of probes at proximal and distal ends of the V_H_ locus, it was also shown for the first time that the *Igh* locus was in a more compacted conformation in recombining B cells. Subsequently, lineage- and developmental stage-specific locus contraction was observed for all of the large AgR loci: *Igκ, Tcrα/δ*, and *Tcrβ* (7–10). This process of locus contraction is reversible, as demonstrated by the extension of the *Igh* locus in pre-B cells, when *Igh* rearrangement is complete (7). Contraction and re-extension of the distal end of the *Tcrα/δ* locus was also observed in double positive (DP) T cells (8). At this locus, contraction is necessary in double negative (DN) T cells for the accessibility of V genes used in TCRδ rearrangements, but in DP thymocytes, rearrangement of the more J-proximal Vα genes occurs before the rearrangement of distal Vα genes, so extension of the distal Vα genes would facilitate the ordered rearrangement of TCR Vα genes.

**Figure 2:**
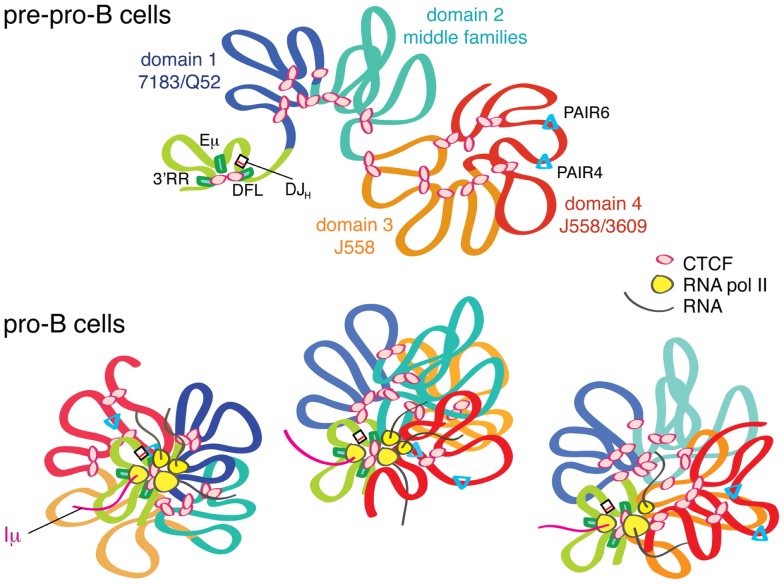
**The *Igh* locus undergoes locus contraction as cells develop from pre–pro-B to pro-B cells**. In pre–pro-B cells, the *Igh* locus is in an extended conformation in a multi-loop rosette structure probably held together by CTCF. In this stage, the D, J, C genes and the enhancers are in one domain that is created by long-range looping of CTCF/DFL and CTCF/3′RR. Eμ also interacts with these two CTCF clusters. This looping creates a D–J domain, which is physically separated from the V_H_ genes, thus facilitating DJ_H_ before V_H_ to DJ_H_ rearrangement. As the cells differentiate into pro-B cells, PAIR elements and other regions within the V_H_ locus start producing RNA transcripts. Through sharing or centralization of transcriptional machinery, a transcription “factory” is formed. This gathering of all of the transcribed regions of the *Igh* locus in a single cell into one location, the transcription factory, will directly result in compaction of the locus because the strong Iμ transcript is constantly produced from Eμ, which is adjacent to DJ_H_. We hypothesize that different regions of the *Igh* locus are transcribed in different cells, and that only a subset of regions are being actively transcribed at any given moment, as depicted by the three pro-B cells in this figure. Thus, in each pro-B cell, different segments of the *Igh* locus are brought into proximity to the rearranged DJ_H_.

Greater insight to how such large-scale locus contraction may occur came from a 3D-FISH study by Jhunjhunwala et al. that used multiple 10 kb probes spanning the entire *Igh* locus followed by 3D computational reconstruction of the location of all the probe binding sites (11). The results showed that the locus could be divided into three ~1 Mb compartments in pre–pro-B cells in which multiple chromatin loops formed rosette-like structures (Figure 2). These compartments then collapsed into a single globule as cells developed into pro-B cells. This brought the distal V_H_ region into closer proximity within 3D space to the DJ_H_ genes and regulatory elements, and in fact the distal V_H_ genes were found to be a similar distance away from the DJ_H_ region as the proximal V_H_ genes (11).

It has been demonstrated that locus contraction of the *Igh* locus is regulated, directly or indirectly, by several key transcription factors. Mice deficient in YY1, Pax5, or the histone methyltransferase Ezh2 were impaired in locus contraction and in the rearrangement of distal V_H_ genes (12–15). Ikaros has also been implicated in *Igh* locus contraction (16), but Rag1/2 is not required for this process (5). Together, these studies suggest that contraction is a pre-requisite state for efficient recombination of distal V_H_ genes. Nonetheless, while AgR locus contraction is well established as a shared process among the large AgR loci that brings distal regions into closer 3D proximity to J genes prior to recombination, it has not been firmly determined what factors may be executing this task in the different lineages.

## CTCF and Cohesin Bind Extensively within AgR Loci

CTCF is an 11 zinc-finger protein that is the only known insulator binding protein in vertebrates (17, 18). Insulators are genetic regions that prevent heterochromatin on one side of the insulator from spreading into the other side. They can also prevent against positional effect variegation, or varied expression of transgenes, depending upon the site of integration in relation to where the insulator is located. Some insulators also have enhancer-blocking activity, where an enhancer cannot activate a promoter when separated by an insulator. It is now known that insulators function through CTCF that creates long-range chromatin interactions by binding to other CTCF bound sites (19). In this way, a domain is created by these chromatin loops, and activity or inactivity of the genes within the domain is insulated from the activity of neighboring domains. In fact, CTCF has been found to play a major role in the establishment of the higher order organization of chromosomes genome-wide, and it is found at the boundaries of topological domains in numerous Hi-C studies (20–22).

CTCF is aided in this domain-creating function by cohesin. Cohesin’s only known function until a few years ago was to hold sister chromatids together during mitosis by forming a ring around the sister chromatids with its four protein subunits (23). Now it is well recognized that cohesin is bound to many active CTCF sites, and thought to reinforce the loops created by the long-range CTCF–CTCF binding (24–26).

Because of the capability of CTCF to form long-range loops, we hypothesized that if CTCF were present at many sites in the AgR loci, it may play a role in determining the 3D structure of the loci and could possibly even influence locus contraction. Thus, we performed ChIP-chip, and subsequently ChIP-seq, to demonstrate that indeed CTCF was bound at numerous sites within the Ig loci, and was therefore an excellent candidate for creating multiple long-range loops (27, 28). If CTCF also had an important role in locus contraction, then we would predict that it would only be bound to the *Igh* locus in pro-B cells, the stage at which the *Igh* locus undergoes contraction. However, we found by ChIP/qPCR that CTCF had a similar pattern of binding in pre-B cells and even in thymocytes, showing that CTCF binding was not lineage- or stage-specific (28). However, widespread binding of CTCF within the *Igh* locus was not observed in fibroblasts, demonstrating that the binding was at least lymphoid-specific. We then analyzed the binding pattern of cohesin by performing a ChIP/qPCR for Rad21, one of the cohesin subunits. This revealed that the level of Rad21 binding was higher in pro-B cells than in pre-B cells or thymocytes for many sites, suggesting cohesin may have a greater role than CTCF in specifying the developmental stage in which *Igh* recombination occurs (28).

CTCF displayed more lineage- and developmental stage-specific binding at the *Igκ* locus (28). Some sites were only bound in pre-B cells, while others showed lower levels of binding in pro-B cells or thymocytes. Rad21 binding also displayed similar lineage and stage-specificity at the *Igκ* locus. Investigation of ChIP-seq of CTCF binding at the large TCR loci showed various extents of lineage- and stage-specificity. At all AgR loci, however, we observed that the binding of cohesin was highest in the appropriate lineage and developmental stage. From these observations, it can be seen that CTCF and Rad21 may have different degrees of function in regulating lineage and stage-specific 3D structures at each AgR locus.

## CTCF and Cohesin Influence the Three-Dimensional Structure of Antigen Receptor Loci

To determine if CTCF made long-range loops that contributed to the compacted structure of the *Igh* locus in pro-B cells, we knocked down CTCF expression in RAG^−/−^ pre-B cells that were cultured in IL7 for 4 days (27). 3D-FISH was performed 4 days after knockdown of CTCF, and the spatial distance between two probes at the far ends of the *Igh* locus did increase, although not to the extent observed in YY1-deficient pro-B cells. This could be due to the fact that while CTCF binding was significantly reduced it was not completely eliminated at the *Igh* locus in the knocked-down pro-B cells as determined by ChIP. However, it is likely that CTCF is only one of many factors that are involved in the compacted structure of the *Igh* locus.

Further insight into the contribution of CTCF to the 3D structure of the *Igh* locus came from the 4C studies of Guo et al. (4). They described two different kinds of loops that formed at the *Igh* locus: Eμ-dependent and Eμ-independent loops. Using a CTCF ChIP-loop assay, they showed that the proximal regions had several CTCF-dependent and Eμ-independent interactions, spanning a region of ~140 kb, as well as interactions with CTCF/DFL. Using a probe in the distal J558 region in the CTCF ChIP-loop assay, they demonstrated four sites of interaction within a 500 kb region, about half of the number of sites seen in 4C with the same distal probe. Importantly, none of the distal CTCF-dependent loops interacted with any other part of the *Igh* locus, and similarly the loops in the proximal region only interacted locally. Jhunjhunwala et al. previously demonstrated that the *Igh* locus consisted of three distinct rosette-like multi-looped structures in pre–pro-B cells that compacted upon themselves during locus contraction (11). Thus, it may be that most of the CTCF-dependent loops described by Guo et al. are local interactions that form the basic rosette-like loops within the *Igh* locus. In addition to CTCF-mediated loops, locus contraction results from further large-scale interactions of these rosettes that are dependent upon Eμ. It may be that the longer range interactions require other key transcription factors such as YY1 and Pax5. YY1 binds to Eμ, and Pax5 binds to PAIR elements, the sites of greatest antisense transcription (29, 30). Whether these are the regions of most importance for YY1 and Pax5 binding with regard to locus contraction, or whether their primary influence is indirect, is not known. Our previous results that showed an increase in spatial distance between the two ends of the *Igh* locus after CTCF knockdown may reflect a loosening of the individual rosette structures while still being held together by other locus contraction regulating factors.

## Insulator CTCF Sites between the V Regions and D/J Genes at AgR Loci Regulate Repertoire Diversity

The *Igh* locus has a pair of CTCF sites 3–5 kb upstream of the last functional D_H_ gene, DFL16.1 (28) (Figure 1). We and others have shown that this pair of CTCF sites (CTCF/DFL) has enhancer-blocking insulator activity in a traditional *in vitro* insulator assay (28, 31). By 3C, we have shown that CTCF/DFL loops to the cluster of nine CTCF sites downstream of the 3′ regulatory region (3′RR) and to Eμ (27), and this was subsequently confirmed by two other groups (4, 32). Coincidently, Jhunjhunwala et al. utilized a probe near CTCF/DFL in their trilateration study (11), so we know that this D_H_ and J_H_ gene containing-loop is located far from the V_H_ genes in pre–pro-B cells, but it moves in close proximity to V_H_ genes in pro-B cells (Figure 2). We hypothesized that this loop creates a domain that contains all the D_H_, J_H_ and constant region genes as well as the Eμ enhancer, but excludes V_H_ genes (27). This would provide a physical environment in which D_H_ to J_H_ rearrangement could occur without any V_H_ genes in the vicinity.

Since the D_H_ genes have much antisense transcription, it was hypothesized that perhaps the function of CTCF/DFL was to stop antisense transcription from extending into the proximal V_H_ genes, preventing accessibility of those V_H_ genes (31). Indeed, deletion of the entire 96 kb intervening region between DFL16.1 and 7183.2.3 resulted in increased levels of D_H_ antisense transcription and extension of this transcription into the proximal V_H_ locus (33). However, knockdown of CTCF in pro-B cells with an intact *Igh* locus only resulted in extension of the antisense transcription for ~4 kb, and the antisense transcription dropped precipitously at the 3′Adam6 gene (27). Thus, preventing D_H_ region antisense transcription from extending into the V_H_ region does not seem to be the function of CTCF/DFL.

Importantly, Guo et al. deleted or mutated the CTCF/DFL sites, and the consequences were profound (32). Ordered rearrangement was perturbed, such that V_H_ to D_H_ rearrangement occurred as well as D_H_ to J_H_ rearrangement. More strikingly, rearrangements were confined to the two most proximal V_H_ genes. This shows that one critical function of these CTCF/DFL sites is to allow the creation of a diverse repertoire of *Igh* rearrangement, fully utilizing all of the V_H_ genes, although the mechanism by which this is achieved is not clear (34). In addition to these striking changes, deletion of CTCF/DFL resulted in a lack of lineage restriction, with V_H_ rearrangement being observed in thymocytes. Thus, two of the basic tenets of the accessibility hypothesis, ordered rearrangements and lineage- and stage-specific restriction of V(D)J rearrangement, are regulated by this pair of CTCF binding sites at CTCF/DFL.

The *Igκ* locus has two pairs of CTCF sites between the Vκ and Jκ genes (28) (Figure 1). One pair is within a region called “Sis” (Silencer in the Intervening Sequence), which also contains several Ikaros binding sites (35). When Garrard and colleagues deleted the 650 bp Sis element in the germline (36), these mice showed a modest preference for rearranging proximal Vκ over distal Vκ genes, and sense non-coding transcription over Vκ genes was also slightly increased. Much more striking was the germline deletion of the strong CTCF sites upstream of Sis in the region called “Cer” (Contracting Element for Recombination) (37). In the Cer^−/−^ mice, sense transcription over a few proximal Vκ genes was increased modestly, but there was a very strong bias toward rearrangement of the most proximal Vκ genes and a great reduction of rearrangement of the remainder of genes. This effect was reminiscent of the strong over utilization of the most proximal V_H_ genes in the CTCF/DFL deletion mice (32). Significantly, some *Igκ* rearrangement was observed in thymocytes in Cer^−/−^ mice (although mainly limited to Jκ1), suggesting that the insulator sequences downstream of the V genes in both *Igh* and *Igκ* loci are major contributors to the lineage restriction of Ig rearrangement. It should be mentioned that the *Igκ* locus contraction was also reduced in Cer^−/−^ mice, meaning extension of the locus could be a reason for the strong bias toward the most proximal V genes. Nonetheless, CTCF/DFL knockout mice did not display any change in *Igh* locus compaction (32), suggesting different modes of repertoire restriction at the two AgR loci.

In addition to the above studies in which the CTCF sites downstream of the V loci have been deleted, CTCF-deficient mice have been studied for their effects on repertoire formation. Hendriks and colleagues examined the *Igκ* locus in mice carrying a B lineage-specific deletion of CTCF (38). By expressing a rearranged *Igh* gene they partially rescued development into pre-B cells. Absence of CTCF in pre-B cells resulted in a strong shift of usage to the most proximal Vκ genes, where most rearrangements occurred at the 10 most proximal genes within the first ~200 kb in the knockout mice. Vκ ncRNA were increased in this region, while the remainder of Vκ ncRNA remained the same. Using Sis as an anchor/viewpoint for 4C-seq, it was demonstrated that the interactions of Sis with the 300 kb proximal region increased significantly. In contrast, iEκ and 3′Eκ viewpoints demonstrated that the enhancer interactions increased with sites up to 1 Mb into the Vκ locus. However, other than a minor decrease of interaction of 3′Eκ with the end of the Vκ locus, the interactions of these three regulatory regions with the distal half of the Vκ locus was unchanged. From these results, it seems that the majority of these long-range interactions between the enhancers or Sis with the distal 2/3 of the Vκ locus are CTCF-independent interactions. Considering that the complete absence of CTCF in the cells gave a similar phenotype as the Cer^−/−^ mice, the predominant effect of CTCF depletion throughout the *Igκ* locus may be primarily due to the absence of CTCF binding at Cer.

As mentioned above, Rad21 (a subunit of cohesin) binds to CTCF sites in the AgR loci when rearrangement occurs (28, 39, 40). Seitan et al. analyzed the role of cohesin in V(D)J rearrangement at the *Tcrα/δ* locus (Figure 1) through the use of Rad21-deficient DP thymocytes (39). Because cells cannot progress through cell division in the absence of cohesin, its role can only be ascertained in cells that do not divide, making DP thymocytes an appropriate cell type to study. They demonstrated that Rad21-deficiency resulted in reduced long-range looping between the CTCF/cohesin sites at TEA, the promoter of the germline transcripts of the 10 most 5′ Jα genes, and Eα that also contains a CTCF/cohesin binding site. They also found an altered pattern of germline transcription in the Jα region and reduced rearrangement to all but the most 5′ Jα genes in these Rad21-deficient mice.

A more detailed analysis of the role of CTCF/cohesin in TCRα rearrangement was performed using CTCF-deficient thymocytes (40). Shih et al. demonstrated by 3C that TEA and Eα strongly interacted in wild type DP thymocytes, weakly in DN thymocytes, and not at all in B cells. TEA and Eα also interacted with several proximal Vα genes and with some Jα genes, predominantly at the 5′ portion of the Jα region. In the *Tcrα/δ* locus, most functional Vα genes have CTCF sites bound adjacent to the promoters, and thus it appears that normally CTCF nucleates a hub of proximal Vα genes, a subset of Jα genes, and the enhancer to create a functional recombination center. This entire hub of interactions was greatly reduced in Eα-deficient DP thymocytes, and thus dependent upon Eα. Deletion of TEA resulted in a shift of the peak of interaction of Eα to the middle Jα genes, likely explaining the previous observations that TEA deletion shifted the predominant rearrangements and germline transcription to the middle Jα genes (41). In contrast to these results in wild type mice, 3C analysis of CTCF-deficient DP thymocytes revealed a reduction in the Eα interactions with TEA, 5′ Jα, and certain 3′ Vα genes, and the level of rearrangement at the *Tcrα* locus was greatly reduced. Strikingly, the CTCF-deficient DP thymocytes showed increased Eα contacts with the *Tcrδ* gene segments that are just upstream of TEA. Thus, it appears that the role of CTCF is to promote Eα interactions with the 3′ Vα and 5′ Jα genes, while discouraging interactions with the intervening *Tcrδ* genes. 3D-FISH experiments demonstrated that the 3′ end of the locus was still contracted in CTCF-deleted DP thymocytes, but 3C results showed that the long-range interactions were reduced for some 3′ Vα genes in DP thymocytes in the absence of CTCF. The level of transcription paralleled the new contacts as TEA-dependent transcription was decreased and transcription of *Tcrδ* genes was increased. Notably, this pattern of altered transcription and 3C contacts paralleled that seen in TEA^−/−^ mice, suggesting that it is the CTCF binding to TEA in WT DP thymocytes that directs Eα to interact with 5′ Vα and 3′ Jα and promotes their transcription and subsequent rearrangement. CTCF binding to TEA also presumably directs Eα to skip over the more proximal *Tcrδ* genes and instead interact with the 5′ Vα genes further away in the locus. In this way, the function of the CTCF-binding region at TEA resembles that of CTCF/DFL and Cer/Sis in that it prevents interactions with the immediately proximal genes, and instead directs interactions to V genes that are further away, allowing the creation of a diverse repertoire of AgR.

## 3D Changes Caused by Non-Coding RNA

For many years we have known that the J and C genes of each AgR locus undergo high levels of non-coding transcription when the locus is undergoing rearrangement (42, 43). In addition, V genes can produce low levels of sense ncRNA (or “germline transcription”) when they are accessible for rearrangement (44). In a few cases it has been demonstrated that these sense ncRNAs begin at the V gene’s promoter and stop shortly after the RSS and presumably this is the extent of most sense ncRNA. More recently, ncRNA in the antisense direction was described, and these ncRNAs are largely intergenic and longer (45). We performed directional RNA-seq of the *Igh* locus, thus defining all of the sense and antisense ncRNA within the locus in pro-B cells (29). Strikingly, there were three major regions of antisense ncRNA, and two minor antisense regions. The three major transcripts began at three of the PAIR elements, PAIR 4, 6, and 11. The 14 PAIR elements, or Pax5 Intergenic Repeat elements, consist of binding sites for Pax5, E2A, and CTCF. These regions have high levels of H3K4me3 and H3ac, as would be expected since they are so highly transcribed (29). The two minor regions of antisense ncRNA were in the proximal J558 region, the site of the originally described antisense RNA (45), and near the J606 genes.

It is now widely accepted that transcription takes place in subnuclear compartments called transcription factories, which are clusters of RNA polymerases (46, 47). Many genes are transcribed within each transcription factory, and often co-regulated genes occupy one together regardless of their genomic distance, and even genes on separate chromosomes may co-localize to the same factory (47, 48). It can be hypothesized that if all *Igh* ncRNA were to be transcribed from the same transcription factory, any regions within the V_H_ part of the *Igh* locus that are being transcribed will of necessity be brought into juxtaposition with Eμ, which contains the promoter of the predominant Iμ germline transcript (29, 49). Iμ is constantly transcribed and located 1–2.2 kb downstream of the J_H_ genes (50). This would mean that any V_H_ genes being transcribed would be close to the DJ_H_ region to which one of the V_H_ genes would ultimately rearrange in each pro-B cell (Figure 2). In support of this hypothesis, we demonstrated by 3C that PAIR4 and PAIR6, the regions of highest antisense transcription within the V_H_ region, directly interacted with Eμ (29). We knew that YY1^−/−^ pro-B cells do not undergo locus contraction or rearrange distal V_H_ genes. With this in mind, we also showed that YY1^−/−^ pro-B cells did not undergo antisense transcription at PAIR elements, and their PAIR elements did not interact with Eμ (29). Thus, it is possible that the lack of antisense ncRNA in the distal V_H_ region of YY1^−/−^ pro-B cells contributes to their lack of both locus contraction and rearrangement of distal J558 genes. We also saw a modest increase in antisense transcription at PAIR elements in CTCF-knockdown in RAG^−/−^ pro-B cells, and 3C analysis showed modestly increased interactions of PAIR and Eμ. This is consistent with the idea that these interactions are taking place in a common transcription factory (27). By 3D-FISH, larger spatial distances between the proximal and distal ends of the *Igh* locus were seen in pro-B cells with CTCF knockdown, suggesting that CTCF is likely assisting in forming multiple loops within the *Igh* locus that “loosen” as its expression is reduced. However, the increase in PAIR–Eμ interactions that we observed with loss of CTCF expression suggests that CTCF is not a major player in the pro-B specific locus contraction process.

## Deep Sequencing of the Igh Repertoire in Pro-B Cells and Bioinformatic Analyses

While it is necessary to understand the effect of individual elements that regulate accessibility and chromatin structure at AgR loci, it is likely that many different factors are acting in concert for efficient production of a diverse repertoire. Recently, our lab and the Oltz lab adopted a bioinformatic approach with a goal to assign weight to the various factors that influence the frequency of rearrangement of individual V genes. To address this aim, we correlated the sequenced repertoires of mouse *Igh* and *Tcrβ* to ChIP-seq data for histone modifications and transcription factor binding and RNA-seq data for ncRNA transcripts (51, 52).

**Figure 3:**
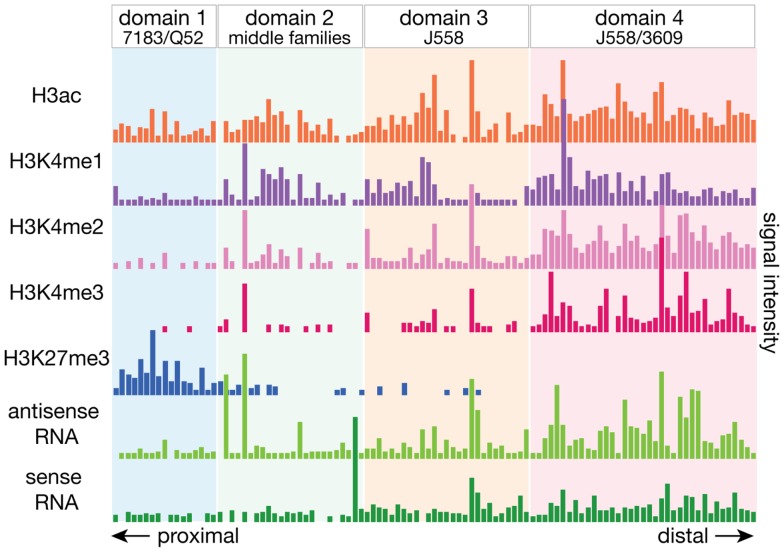
**The *Igh* locus can be divided into four domains by the epigenetic and transcriptional landscape**. The local epigenetic and transcriptional environment of each gene is plotted, with the numbers deriving from the total number of ChIP-seq or RNA-seq reads for the 2.5 kb region centered around each V_H_ gene. Active histone modifications and ncRNA transcripts were enriched at V_H_ genes at the distal end of the locus while proximal genes had very little of these features. Domains were divided by the boundary of V_H_ gene families, and bioinformatic analyses of the various epigenetic elements suggest that genes in each domain may be regulated by different mechanisms.

For the analysis of the mouse Igh repertoire in C57BL/6 mice, we sequenced 5′RACE-amplified cDNA from cell sorter purified pro-B cells to determine the pre-selection repertoire (51). Because this approach utilizes universal sequences to the 5′ annealed adapter and Cμ on the expressed heavy chain transcript, it allows for an unbiased amplification of the expressed repertoire. In pro-B cells, as expected, the V_H_ genes were recombined at widely different frequencies throughout the locus. We assessed the histone post-translational modifications and transcript levels over each actively recombined gene and observed a significant distinction between V_H_ genes at the distal and proximal parts of the locus (Figure 3). Distal J558 family genes had greater enrichment for the active histone modifications (H3K4 methylation and H3 acetylation) as well as higher levels of both sense and antisense transcripts, than the proximal 7183 and Q52 families. This difference in epigenetic profiles suggests that these factors may be preferentially more influential at the distal half of the large *Igh* locus. We therefore divided the *Igh* locus into four domains based on V_H_ gene family locations, and found that domain 1, consisting of the 7183 and Q52 families, had very low levels of H3K4 methylation and the lowest levels of ncRNA. Domain 4, the most distal, containing all of the 3609 family as well as half of the J558 genes, had the highest levels of all the active histone modifications as well as the highest levels of both sense and antisense ncRNA. Domain 3, containing the remainder of the J558 genes, also had active chromatin marks and higher levels of ncRNA than the proximal genes.

When the relation to CTCF and Rad 21 binding was examined, all but one actively utilized gene of the proximal 7183 and Q52 families in domain 1 had a CTCF binding site within 100 bp, and all but one inactive gene had a CTCF site at ~1–20 kb distance. While at a genomic scale, a distance of 100 bp vs. >1 kb may not be of great difference, it may be enough distinction to place an RSS in close enough vicinity to the recombination center at the J_H_ region to provide a significant advantage to a V_H_ gene. CTCF binding at the base of the loop at CTCF/DFL, which is proximal to the rearranged DJ_H_, and the base of the loop of functional V_H_-adjacent CTCF sites in domain 1 would bring these regions in close proximity. Genes in the middle and distal regions did not show this tendency, suggesting that having a close CTCF binding site is most important for the genes at the proximal end of the *Igh* locus.

We previously demonstrated that RSS quality could influence V_H_ gene rearrangement frequency, and demonstrated that three different prototypic 7183 RSSs and a S107 RSS were more effective than a J558 RSS (53). All of the J558 RSSs are much further from the consensus RSS sequence than the 7183 RSSs. However, we also showed that other parameters can override this effect, and that V genes with an identical RSS can rearrange at very different frequencies *in vivo* (53–55). Results from a computational model-building algorithm using our ChIP-seq, RNA-seq, and *Igh* repertoire deep sequencing data determined that having a functional RSS and an open chromatin environment as assessed by histone modifications were significant factors in predicting the activity of a V_H_ gene (51). When just the actively rearranging functional V_H_ genes were considered, the different domains of the V_H_ locus had different factors that correlated with recombination frequency. Within the proximal domain 1, proximity to the DJ_H_ genes was most significant, which is in agreement with the data we obtained a decade ago on another *Igh* haplotype, *Igh^a^*, in pro-B cells from μMT mice (53). In contrast, at the distal domains, higher levels of active histone modifications appeared to be most important. This greater enrichment for active histone modifications at the distal V_H_ genes may reflect recruitment of these genes to the recombination center via transcription or some unknown factor that compensates for the disadvantages such as the distance from the DJ_H_ genes and their poorer RSSs.

At the *Tcrβ* locus, Gopalakrishnan et al. took a different approach of assessing individual Vβ gene usage by using a Taqman assay to measure rearrangement of genomic DNA rather than the 5′RACE approach that we used for the *Igh* repertoire (52). This approach is feasible at the *Tcrβ* locus due to the much smaller number of V genes compared to the *Igh* locus. When recombination frequency was compared to 3C interaction data, there was no rearrangement advantage observed for Vβ genes that displayed higher levels of interaction with the Dβ1 gene, leading authors to conclude that once the contraction has occurred at the relatively smaller *Tcrβ* locus, spatial access is not a determining factor for Vβ gene usage. However, it should be noted that all but two of the Vβ genes are present within 235 kb at this locus, whereas the *Igh* and *Igκ* V genes are spread over >2.5 kb. Therefore, proximity of V genes to (D)J genes in 3D space is much more likely to contribute to V gene rearrangement frequency in the large *Igh* and *Igκ* loci. The bioinformatic analysis of all of the chromatin modifications, transcriptional activity, and 3D proximity for the *Tcrβ* locus led to the conclusion that having a functional RSS, higher nucleosome depletion (FAIRE assay), and higher RNA pol II binding were good indicators for active vs. inert Vβ genes. They also concluded, for the actively rearranging genes, higher levels of active histone modifications correlated with higher levels of recombination, similarly to our conclusions for the domain 3 and 4 V_H_ genes.

The results from the *Tcrβ* and *Igh* locus considered together suggest that while generally accessible chromatin conformation and functional RSS sequences are both important, the different AgR loci are not governed by the same rules. In the case of the *Igh* locus, even the proximal and distal ends of the locus may be regulated by different mechanisms, which is likely due to its great expansion over a large genomic area and hence a greater need for locus contraction to bring the distal and middle V_H_ genes closer.

## Model for the Role of CTCF and ncRNA in the Establishment of the 3D Structure of the AgR Loci

CTCF and its partner cohesin play important structural roles in creating large domains throughout the entire genome. Within AgR loci, there is a much higher density of CTCF/cohesin sites at rearranging loci than elsewhere in the genome. We hypothesize that the many CTCF/cohesin sites are necessary to create the multi-looped rosette-like structure that is the basic conformation of all AgR loci. This rosette structure makes it easier to compact various loci at the time of rearrangement. For some V genes, such as the V_H_ genes in domain 1 of the *Igh* locus, having a CTCF site near the RSS appears to be critical for a V_H_ gene to undergo rearrangement, but these V_H_ genes are rather poor in active histone marks and ncRNA. Thus, in lieu of these accessibility factors, being physically tethered to the recombination center, presumably by interactions with CTCF/DFL, is of great importance. In addition to the many CTCF sites throughout the large V gene portions of the AgR loci, CTCF/cohesin sites in between the V and J regions of the large AgR loci seem to be particularly important in regulating proper V gene rearrangements in a lineage- and developmental stage-specific manner (Figure 1). We also propose that ncRNA, or germline transcription, can directly facilitate *Igh* locus compaction if V_H_ genes or intergenic regions being transcribed are located in the same transcription factory as the Iμ ncRNA. Since the DJ_H_ rearrangement is directly adjacent to the highly transcribed Iμ, transcription will place the DJ_H_ rearrangement very close to the transcription factory. We hypothesize that the structure of the *Igh* locus is very dynamic in pro-B cells, with different subsets of V_H_ genes being transcribed in each pro-B cell (Figure 2, bottom). Thus, we suggest that the dynamic and stochastic nature of germline transcription will physically move different parts of the V_H_ gene locus into proximity to the DJ_H_ rearrangement in each pro-B cell, and this will provide equal opportunity for V_H_ genes throughout the locus to come into proximity to the DJ_H_ rearrangement. Presumably, this same activity could take place at the other AgR loci. In this way, the production of diverse repertoires of antibodies and TCR is assured.

## Conflict of Interest Statement

The authors declare that the research was conducted in the absence of any commercial or financial relationships that could be construed as a potential conflict of interest.
